# Evaluation of validity and reliability of video analysis and live observations to assess trauma team performance

**DOI:** 10.1007/s00068-022-02004-y

**Published:** 2022-07-11

**Authors:** Oscar E. C. van Maarseveen, Wietske H. W. Ham, Stijn van Cruchten, Rauand Duhoky, Luke P. H. Leenen

**Affiliations:** 1 Department of Trauma Surgery, University Medical Center Utrecht, Heidelberglaan 100, 3584 CX Utrecht, The Netherlands; 2 Emergency Department, University Medical Center Utrecht, Heidelberglaan 100, 3584 CX Utrecht, The Netherlands; 3 Institute of Nursing Studies, University of Applied Science, Heidelberglaan 7, 3584 CS Utrecht, The Netherlands

**Keywords:** Resuscitation, Trauma team, Live observers, Video review, Quality assessment

## Abstract

**Introduction:**

A trauma resuscitation is dynamic and complex process in which failures could lead to serious adverse events. In several trauma centers, evaluation of trauma resuscitation is part of a hospital’s quality assessment program. While video analysis is commonly used, some hospitals use live observations, mainly due to ethical and medicolegal concerns. The aim of this study was to compare the validity and reliability of video analysis and live observations to evaluate trauma resuscitations.

**Methods:**

In this prospective observational study, validity was assessed by comparing the observed adherence to 28 advanced trauma life support (ATLS) guideline related tasks by video analysis to life observations. Interobserver reliability was assessed by calculating the intra class coefficient of observed ATLS related tasks by live observations and video analysis.

**Results:**

Eleven simulated and thirteen real-life resuscitations were assessed. Overall, the percentage of observed ATLS related tasks performed during simulated resuscitations was 10.4% (*P* < 0.001) higher when the same resuscitations were analysed using video compared to live observations. During real-life resuscitations, 8.7% (*p* < 0.001) more ATLS related tasks were observed using video review compared to live observations. In absolute terms, a mean of 2.9 (during simulated resuscitations) respectively 2.5 (during actual resuscitations) ATLS-related tasks per resuscitation were not identified using live observers, that were observed through video analysis. The interobserver variability for observed ATLS related tasks was significantly higher using video analysis compared to live observations for both simulated (video analysis: ICC 0.97; 95% CI 0.97–0.98 vs. live observation: ICC 0.69; 95% CI 0.57–0.78) and real-life witnessed resuscitations (video analyse 0.99; 95% CI 0.99–1.00 vs live observers 0.86; 95% CI 0.83–0.89).

**Conclusion:**

Video analysis of trauma resuscitations may be more valid and reliable compared to evaluation by live observers. These outcomes may guide the debate to justify video review instead of live observations.

## Introduction

With the implementation of trauma systems, severe trauma patients are resuscitated with a systematic approach. The initial management of severely injured patients is performed by several (para) medical healthcare professionals and extends over a series of sequential processes that begin with the first responder on the accident site and ends with definitive treatments such as surgery or intensive care at the hospital.

This interdisciplinary response to injuries has resulted in significant improvements in mortality and morbidity of severely injured patients [1–3]. Resuscitation by a trauma team is one of the cornerstones of a structured response to injury, especially for severely injured patients [4–6]. The objective of a trauma team is to assess all life-threatening injuries in patients and offer immediate resuscitation and stabilization if necessary.

A trauma resuscitation is a dynamic process, where several tasks are performed simultaneously or in quick succession. During these resuscitations, the Advanced Trauma Life Support (ATLS) guidelines, which are acknowledged worldwide, provide guidance to the trauma team by prioritizing diagnostic and therapeutic processes [7]. Nevertheless, during this dynamic process, failures in technical or non-technical skills (e.g., communication or leadership) could cause serious adverse events, and even mortality [8–11]. Therefore, there is a continuous effort to optimize healthcare for severely injured patients. To this end, the American College of Surgeons requires quality assessment programs to be certified as a level one trauma center [12];hence, several trauma centers have incorporated evaluation of trauma team resuscitations within their quality assessment program [13, 14].

The validity and reliability of trauma team evaluations are crucial, as these evaluations are the foundation upon which quality assessments and education are built. Video analysis has been described as an effective approach to assess trauma team performance, as it provides an accurate documentation of resuscitation. For example, the ability to replay a video allows for a detailed analysis of (non-)technical skills and processes during the resuscitation [16–22]. However, privacy issues and regulations withhold several trauma centres from using video to review trauma resuscitations [22]. Due to the these medicolegal issues, some hospitals use live observers instead of video analysis to asses trauma team performance [23, 24]. A systematic comparison of the validity and reliability of video analysis and live observations as methods to assess trauma team performance is lacking. The aim of this study was to compare the validity and reliability of video analysis and live observation as methods to evaluate trauma resuscitations on ATLS adherence in simulated and real-life witnessed trauma resuscitations.

## Materials and methods

### Design

**Fig. 1:**
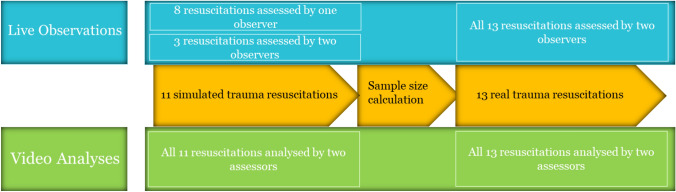
Overview of study design

This study was a prospective observational study to compare the validity and reliability of life observations versus video analysis as methods to assess ATLS adherence in simulated and real-life trauma resuscitations. Audio recordings were also included in the video recorded (simulated) resuscitations. First, eleven simulated trauma resuscitations were assessed. Three simulated resuscitations were assessed by two live observers and eight additional simulated resuscitations were assessed by one live observer. Next, all eleven simulated resuscitations were assessed by two observers by using videos (video analysis). Additionally, thirteen real-life resuscitations were assessed. All real-life resuscitations were assessed by two live observers and two video assessors (Fig. 1). Throughout the study period, the video assessors were the same investigators as the live observers and blinded for each other’s findings. The investigators were not familiar with the trauma team members. Team members’ roles are identified by the colour of their lead apron. All resuscitations were assessed using a predefined list of twenty-eight ATLS related tasks (Table 1). The same list was used during both simulated and real-life resuscitations by live observers and by the video assessors.

**Table 1:** ATLS adherence

ATLS related task	Simulated resuscitations^a^				Real life resuscitations^b^			
	Observers (%)	Video (%)	Difference (%)	*p* Value	Observers (%)	Video (%)	Difference (%)	*p* Value
Total observed ATLS tasks	63.0	73.4	10.4	< 0.001*	55.4	64.1	8.7	< 0.001*
Airway assessment	90.9	100	9.1	0.090	92.3	92.3	0.0	–
Intubation	54.5	63.7	9.2	0.069	23.1	23.1	0.0	–
Rigid collar	90.9	90.9	0.0	0.560	61.5	61.5	0.0	–
Headblocks	100	100	0.0	–	69.2	69.2	0.0	–
Breathing assessment	100	100	0.0	–	88.5	100	11.5	0.083
Chest tube	68.2	72.7	4.5	0.946	0.0	0.0	0.0	–
Needle thoracentesis	13.7	18.2	4.5	0.816	0.0	0.0	0.0	–
Oxygen administration	100	100	0.0	–	50.0	53.8	3.8	0.327
Pulse oximeter	77.3	100	22.7	0.035*	88.5	100	11.5	0.083
First IV line	100	100	0.0	–	80.8	100	19.2	0.022*
Second IV line	81.9	100	18.1	0.090	26.9	53.8	18.9	0.006
Fluid administration	77.3	100	22.7	0.035*	76.9	100	23.1	0.011*
Withdrawal of blood samples	100	100	0.0	–	95.2	100	4.8	0.327
Results of arterial blood gas	68.2	100	31.8	0.005*	61.5	69.2	7.7	0.161
EKG monitor	68.2	90.9	22.7	0.020*	69.2	100	30.8	0.003*
Order of blood products	59.1	86.6	27.5	0.031*	0.0	7.7	7.7	0.161
Arrival of blood products	31.9	59.1	28.0	0.014*	0.0	0.0	0.0	–
Blood pressure and heart rate	81.9	100	18.1	0.090	100	100	0.0	–
Pelvic examination	81.9	81.9	0.0	–	84.6	92.3	7.7	0.077
Abdominal examination	90.9	100	9.1	0.239	92.3	100	7.7	0.077
Long bone examination	45.5	77.3	31.8	0.023*	76.9	84.6	7.7	0.077
Pupil examination	9.1	18.2	9.1	0.390	42.3	88.5	46.2	0.077
Neurological examination	9.1	18.2	9.1	0.354	88.5	92.3	3.8	0.038
Log roll	9.1	18.2	9.1	0.534	46.2	46.2	0.0	–
Temperature measurement	9.1	18.2	9.1	0.584	3.8	23.1	16.3	0.192
Introduction of catheter	0	9.1	9.1	0.249	30.8	30.8	0.0	–
Warm Blankets	86.4	100	13.6	0.090	100	100	0.0	–
Rectal exam	0.0	0.0	0.0	–	0.0	7.9	7.9	0.077

^*^Significant *p* ≤ 0.05, ^a^overlapping *t* test; ^b^student *t* test

### Setting and sample

The study was performed at the University Medical Center of Utrecht, a level one trauma center and a Joint Commission International (JCI) accredited tertiary care facility in the Netherlands. The trauma team training took place in the same resuscitation room where patients were resuscitated, while the simulated trauma resuscitations were weekly yielded using the SimMan^®^. During each training session, a scenario of a severely injured patient was simulated. The trauma team composition during simulation was comparable to the composition during real-life resuscitations, only without a neurologist and radiology technician; a review by Kreb et al. [20] contains a detailed description of the trauma team’s composition and the trauma team activation criteria. Thirteen consecutive real-life resuscitations of adult injured patients resuscitated with a trauma team were assessed.

### Outcomes

#### Validity

Validity is defined as the extent to which a measurement method or test measures what it is supposed to measure. An assumption of the study was that only false negative observations would occur and there would be no false positive observations, meaning that tasks would be missed by the live observers or by using video analysis, but no tasks would be observed that did not occur. Therefore, the total number of observed ATLS tasks was our primary outcome measure to assess validity. The total observed ATLS tasks was defined as observed tasks divided by the total number of listed ATLS related tasks which should be performed (28 tasks) [7]. The total observed tasks were represented in percentages. The second validity assessment was to compare the observed separate ATLS related tasks between life observers and video assessors.

#### Reliability

Reliability is defined as the extent to which a repeated measurement method provides the same results. Our primary outcome to assess reliability was interobserver variability of live observations and video analysis for the observation of ATLS-related tasks during simulated and real-life resuscitations. Interobserver variability is be defined as the degree of agreement among observers.

### Sample size calculation

G-power was used to calculate the needed sample size for the real life resuscitations. [25] The results of the video analysis and live observations of simulated resuscitations were used to estimate the sample size for the real-life resuscitations. As the assessment of simulated and real-life resuscitations are likely to differ in practice, a 50% deviation from the effect size found during simulated resuscitations was used to calculate the needed sample size of real-life resuscitation. Within G-power, in the family of *t* tests, a 2-tailed matched pairs test was used to calculate the total sample size. The calculated sample size was 13, which was based on the *α* error of 0.05, the power (1-*β* error) of 0.95, and the effect size of 1.1.

### Statistical analysis

#### Validity

Differences in overall observed ATLS adherence during real life resuscitations between live observers and video analysis were compared using the Student’s *t* test using SPSS (IBM Corp. Released 2012. IBM SPSS Statistics for Windows, Version 21.0. Armonk, NY: IBM Corp). Differences in overall observed ATLS adherence between live observers and video analysis during simulated resuscitations were compared using the partially overlapping *t* test as described by Derrick et al. [26] using R-studio, whereas there are within both groups paired and unpaired observations. A *p* value of less than 0.05 was deemed statistically significant.

#### Reliability

Interobserver variability was calculated using the intra-class correlation coefficient (ICC) using SPSS (IBM Corp. Released 2012. IBM SPSS Statistics for Windows, Version 21.0. Armonk, NY: IBM Corp.). A higher ICC value indicates a higher level of agreement among the ratings. Perfect agreement is shown by an ICC value of 1.0, while random agreement is indicated by a value of zero, and a pattern of systematic disagreement is shown by negative ICC values. The cut-off points for qualitative ratings of agreement based on ICC values were based on the article by Cicchetti et al. [27] where interobserver reliability is considered low for ICC values less than 0.40, fair for ICC values between 0.40 and 0.59, good for ICC values between 0.60 and 0.74, and excellent for ICC values between 0.75 and 1.0. Differences in ICC were deemed significant in case 95% confidence interval (CI) did not overlap.

### Standards ethical statement

The Medical Ethical Committee of the University Medical Center Utrecht approved conduction of this study and have therefore been performed in accordance with the ethical standards laid down in the 1964 Declaration of Helsinki and its later amendments Thereby, as agreed with the hospital’s legal department, no informed consent from patients or workers was required because the institution uses video registration as part of local quality assessments. Videos of resuscitation were stored on a server, separately from patient record databases. To protect the privacy of patients and employees, all captured videos were analysed within and automatically deleted after 30 days. No patient related data was gathered for this study. Finally, the authors have nothing to declare and have no conflict of interest.

## Results

Eleven simulated resuscitations and thirteen real life resuscitations were live observed and reviewed on video.

### Validity

Table 1 shows the total observed ATLS-related tasks using video analysis and live observations of simulated and real-life resuscitations. Overall, the percentage of observed ATLS tasks was 10.4% (*p* < 0.01) higher when resuscitations were assessed using video analysis compared live observations in simulated resuscitations, and 8.7% (*p* < 0.01) higher when resuscitations were assessed using video analysis compared to live observations in real-life resuscitations. In absolute terms, 2.9 (during simulated resuscitations) respectively 2.5 (during actual resuscitations) ATLS-related tasks per resuscitation were not identified using live observers, that were observed through video analysis. During simulated resuscitations, twenty-one of the twenty-eight ATLS related tasks were more often observed using video analysis compared to live observations. Of these eight tasks were significantly more observed using video review compared to live observation: pulse oximeter (100 vs. 77.3%; *p* = 0.035), fluid administration (100 vs. 77.3%; *p* = 0.035), announcing results of arterial blood gas (100 vs. 68.2%; *p* = 0.005), EKG monitoring (90.9 vs. 68.2%; *p* = 0.020), order of blood products (86.6 vs. 59.1%; *p* = 0.031), arrival of blood products (59.1 vs. 31.9%; *p* = 0.014) and long bone examination (77.3 vs. 45.5%; *p* = 0.023). During real-life resuscitations, eighteen ATLS related tasks were more often observed using video analysis compared to live observations, of which four were significant: first intravenous line (100% vs. 80.8%; *p* = 0.022), second intravenous line (53.8 vs. 26.9%; *p* = 0.006), EKG monitoring (100 vs. 69.2%; *p* = 0.003) and neurological examination (92.3 vs. 88.5%; *p* = 0.038).

### Reliability

Interobserver variability for assessing adherence to ATLS related tasks was significantly higher when assessed using video analysis versus live observations for both simulated resuscitations [ICC 0.97 (0.97–0.98) vs. 0.69 (0.57–0.78)] and real-life resuscitations [0.99 (0.99–1.00) vs. 0.86 (0.83–0.89)] (Table 2).

**Table 2:** inter class reliability

	ICC live observations (95% CI)	ICC video review (95% CI)
Simulated resuscitations		
ATLS tasks	0.69 (0.57–0.78)	0.97 (0.97–0.98)
Real resuscitations		
ATLS tasks	0.86 (0.83–0.89)	0.99 (0.99–1.00)

## Discussion

This is the first study to compare validity and reliability of live observations and video analysis to evaluate trauma resuscitations on ATLS adherence. From our study results, video analysis appears to be more valid compared to live observations, as significantly more ATLS related tasks were observed. Furthermore, the degree of agreement using video analysis was significantly higher compared to live observers. Superiority of video analysis over live observations of real-life resuscitations have been seen in previous studies for the evaluation of non-technical skills. Reliability of the T-NOTECHS, a tool to assess non-technical skills, was measured using video analysis [22]. In that study, we found an ICC of 0.94 and 0.84, respectively, when reliability during real life resuscitation was measured as the mean of three assessors or a single assessor [22]. In the study by Steinemann et al. [23] the interobserver variability of non-technical skills assessment during simulated resuscitations was higher using video analysis (ICC 0.71) compared to assessment of live observers (ICC 0.44). Furthermore, in the study by House et al. [28] the performance of emergency medicine residents during pediatric rapid sequence induction of anesthesia and intubation were assessed by live assessors and video analysis. In their study, overall interrater agreement for video analysis was higher compared to live observations. (ICC 0.79 vs ICC 0.75).

A key implication of the results is that video analysis might be more appropriate for ongoing quality assurance programs in level one trauma centers compared to live observations. In a recent nationwide survey across United States’ level one and two trauma centers, 65% of respondents reported that video analysis resulted in performance improvement initiatives, 24 and 41% stated that video analysis has led to changes in institutional guidelines. [24] However, medicolegal and patient privacy concerns were expressed as main barrier to implement video review of trauma resuscitation. [24] Interestingly, only 2.8% of trauma centers had first-hand experience with a video analysis-related medical-legal problem. Moreso, video review may even reduce medicolegal cases, as Yang et al. [29] found a significant relation between patient safety and the risk of medicolegal involvement of physician in Canadian hospitals. In other words, video review may enhance patient safety, which may result in less medicolegal issues of physicians. Thereby, live observations should not be assumed to be less incriminating compared to video reviews. One should seek legal counsel before implementing a quality assessment program of trauma team resuscitation.

To mitigate privacy threats, proper informing, security, and anonymization methods should be adopted while performing video analysis. Quality improvements through video assessment should be secure and anonymized, and personnel should be informed being video using a clear sign at the entrance of the emergency and updated using local hospital information platforms. Data should be stored securely and must comply with local regulations, and access to the videos should be restricted to only a few key personnel. All of these actions should be well-documented and regularly evaluated. Finally, there are some recently described advanced methods available that could significantly ano[nymize] patients and personnel in the trauma room. In the study by Silas et al. (29) videos of operating rooms during surgery were processed into point clouds. Recognition of staff by their colleagues was rated using a Likert scale, where the score of 1 was anonymous, (unable to identify) and a score of 10 was not anonymous, (easy to identify) The mean scores for unaltered and point cloud videos were 7.05 and 1.41, respectively (*p* < 0.001). Thereby, the authors noted that evaluation of surgical activity was still possible using this method.

### Strengths and limitations

Our study methodology included a sample size calculation, and was able to sufficiently demonstrate differences in reliability and validity between video analysis and live observations to assess the adherence of ATLS related tasks. Another strength of this study is that both real life trauma resuscitation and simulated resuscitation were evaluated. However, there are limitations to our research that should be considered. First, recall bias may have been introduced, as the life observers were the same persons as the video assessors. Therefore video assessors may have remembered some parts of the resuscitation as they also have witnessed the same resuscitation in real life. This effects is tried to minimalize as, the videos are assessed 5 till 30 days after the resuscitation occurred. A longer period between the actual resuscitation and is not possible as videos were removed within thirty days due to local hospital security and privacy policies. Second, this study assumed only false negative observations and no false positive observations, implying that live observers or video analysis would miss tasks, but no tasks would be observed that did not occur. However, false positive observations are theoretically possible and are more likely to occur during live observations compared to video review. A assessor could reviewed parts of the resuscitation multiple times during video analysis in case the assessor has reservations about a specific activities, which is not possible during live observation. By assumption no false positive observations would occur, the assessment of this study was limited to whether the task was performed or not, and did not evaluate whether it was performed well or not. However, in this study, no ATLS-related tasks were identified by live observers that were not found using video analysis, indicating that the chance of false positive observations appears low. Third, the interrater variability of live observers for simulated resuscitations should be interpreted with caution. Only three of the eleven simulated resuscitations were assessed by two live observers, which means that analysis of interobserver variability for live observers in simulated setting included only three cases. Therefore, values found for interobserver variability of live observers in simulated resuscitations are more uncertain than interobserver variability of video analysis or live observations during real life resuscitations, which are reflected in the confidence intervals. Finally, no patient related data was gathered; therefore, we were not able to take severity of injury into account. Resuscitations of severely injured patients are more dynamic, and may therefore be challenging to observe for live observers, while in contrast, video assessment creates the opportunity to replay a video which may even increase reliability of the assessment in these resuscitations. Therefore, we strongly advise to use video analysis to assess trauma resuscitations.

## Conclusion

Video analysis of trauma resuscitations may be more valid and reliable compared to evaluation by live observers. These important outcomes may guide the debate to justify video review instead of live observations, albeit with possible ethical concerns. Future work should evaluate ways to overcome the ethical issues in order to provide a more efficient way of analyzing and retaining trauma resuscitation procedures.
